# Perineuronal Nets in the CNS: Architects of Memory and Potential Therapeutic Target in Neuropsychiatric Disorders

**DOI:** 10.3390/ijms25063412

**Published:** 2024-03-18

**Authors:** Xue Li, Xianwen Wu, Tangsheng Lu, Chenyan Kuang, Yue Si, Wei Zheng, Zhonghao Li, Yanxue Xue

**Affiliations:** 1 National Institute on Drug Dependence, Peking University, Beijing 100191, China; lx@bjmu.edu.cn (X.L.); lutsh@stu.pku.edu.cn (T.L.); siyue@bjmu.edu.cn (Y.S.); lzhongh@hsc.pku.edu.cn (Z.L.); 2 School of Basic Medical Sciences, Peking University Health Science Center, Beijing 100191, China; 3 Department of Laboratory Animal Sciences, Peking University Health Sciences Center, Beijing 100191, China; wuxianwen@bjmu.edu.cn; 4 College of Forensic Medicine, Hebei Key Laboratory of Forensic Medicine, Collaborative Innovation Center of Forensic Medical Molecular Identification, Hebei Medical University, Shijiazhuang 050017, China; kcy58264@163.com; 5 Peking-Tsinghua Centre for Life Sciences, PKU-IDG/McGovern Institute for Brain Research, Peking University, Beijing 100871, China; zhengw@stu.pku.edu.cn

**Keywords:** perineuronal nets, parvalbumin, plasticity, memory, neuropsychiatric disorders

## Abstract

The extracellular matrix (ECM) within the brain possesses a distinctive composition and functionality, influencing a spectrum of physiological and pathological states. Among its constituents, perineuronal nets (PNNs) are unique ECM structures that wrap around the cell body of many neurons and extend along their dendrites within the central nervous system (CNS). PNNs are pivotal regulators of plasticity in CNS, both during development and adulthood stages. Characterized by their condensed glycosaminoglycan-rich structures and heterogeneous molecular composition, PNNs not only offer neuroprotection but also participate in signal transduction, orchestrating neuronal activity and plasticity. Interfering with the PNNs in adult animals induces the reactivation of critical period plasticity, permitting modifications in neuronal connections and promoting the recovery of neuroplasticity following spinal cord damage. Interestingly, in the adult brain, PNN expression is dynamic, potentially modulating plasticity-associated states. Given their multifaceted roles, PNNs have emerged as regulators in the domains of learning, memory, addiction behaviors, and other neuropsychiatric disorders. In this review, we aimed to address how PNNs contribute to the memory processes in physiological and pathological conditions.

## 1. Introduction

In the intricate milieu of the brain, the extracellular matrix (ECM) serves as a pivotal piece of architecture, orchestrating both physiological processes and pathological aberrations [1,2]. Among the ECM’s specialized structures, perineuronal nets (PNNs) are reticular structures composed of aggregations of ECM molecules. Ubiquitous across the mammalian central nervous system’s (CNS) cells, PNNs mostly surround the soma and dendrites of GABAergic neurons, particular the fast-spiking parvalbumin interneurons (PV cells). The architecture provides both a physical barrier and an anionic shield which preserves the integrity of synaptic junctions and insulates them from potentially damaging neurochemical stimuli. PNNs have been shown to define the critical period plasticity and early neural trajectories, influencing processes from neuron migration and differentiation to axonal guidance, especially within visual and motor systems [3,4]. An intriguing temporal dynamic characterizes PNNs, with their emergence occurring in a particular experience-dependent manner during postnatal stages, reaching its expression peaks in adulthood, while being conspicuously minimal in newborn and aged animals [5]. Pizzorusso offered seminal insight by highlighting PNNs as gatekeepers in determining the closure of the critical period for ocular dominance plasticity [2]. Interestingly, this experience-dependent plasticity associated with PNNs could be reinitiated in adulthood by removing PNNs enzymatically [2]. It is also worth noting that synaptic plasticity underpins memory throughout the life’s critical junctures, from developmental stages to learning epochs, and post exposure to drug abuse, as well as during the aging process [6,7,8,9]. Alterations in PNNs invariably influence the synaptic framework, manifesting in memory, behaviors, and even susceptibility to psychiatric conditions [10,11,12,13]. An accumulating number of findings suggest PNNs as a key regulator for learning, memory, and information processing both in healthy individuals and in a plethora of pathological conditions, including brain damage, Alzheimer’s disease (AD), epilepsy, autism, and drug addiction [14]. To date, many studies of PNNs have focused on their role in plasticity. However, how PNNs affect memory by regulating synaptic plasticity is not fully understood. In this review, we will briefly describe the composition and physiological function of PNNs, and then dissect the multifaceted roles of PNNs in the mammalian CNS, casting a spotlight on their influence across the spectrum of memory and memory-related dysfunctions, from the conditions of spatial memory, fear memory, drug addiction, and neurodegeneration. Deciphering the nexus between PNNs, synaptic plasticity, and memory may lead to therapeutic intervention against neurodegenerative and neuropsychiatric disorders, including PTSD, AD, addiction, and age-associated cognitive impairments.

## 2. PNNs’ Structure and Functions

### 2.1. Composition and Distributions

**Figure 1:**
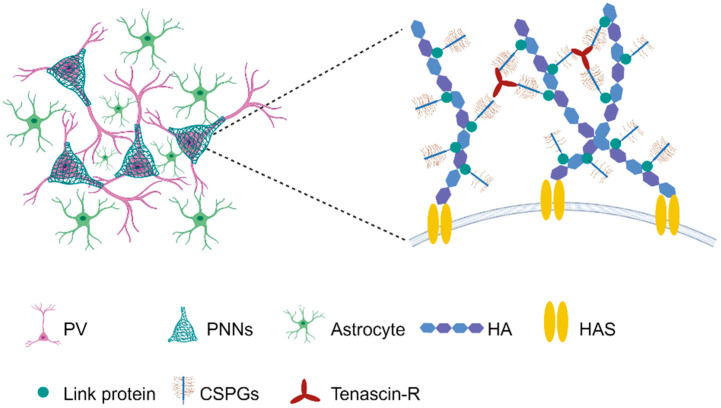
Structure and composition of PNNs. The hyaluronan (HA) forms a mesh-like structure to which other PNN molecules can bind. HA is attached to the cell surface through hyaluronan synthase (HAS). The chondroitin sulfate proteoglycans (CSPG) components, including lecticans, neurocan, versican, and aggrecan are all able to attach to chains of HA through link proteins. Tenascin-R can conjugate up to three CSPG, enhancing the overall rigidity of the PNNs.

Approximately 10–20% of the brain volume is occupied by the ECM, which is a dense network of proteins and glycans that provides anchorage points for nerve and glial cells and contributes to their normal physiology [15]. The cerebral ECM has been recognized as a key player in synaptic plasticity, especially regarding the structures of PNNs. PNNs were first described as reticular structures by Golgi in the late 1800s [16], and since then their appearances has been confirmed across a wide range of species, from frogs and birds to mammals, including humans [17]. The nets are intricate assemblies of highly diverse proteoglycans (PGs) and chondroitin sulphate glycosaminoglycan chains (CS-GAG) attached to chondroitin sulfate proteoglycans (CSPG). These components are anchored on a foundation of hyaluronan (HA) glycosaminoglycan backbones and proteins, which condense through specific interactions around certain neurons [11,12]. Although PNNs in the adult CNS are generally stable structures, various endogenous and exogenous stressors are able to cause their breakdown. For example, matrix metalloproteases (MMPs), produced by various cell types, are involved in remodeling the PNNs and, when disrupted, indiscriminately destroy laminin, collagens, and CSPG [18]. Therefore, the function and stability of PNNs are influenced by the constituent of CS-GAGs, which consist of repeating disaccharide units of sulfated glucuronic acid (GlcA) and N-acetylgalactosamine (GalNac) [11]. The PGs interact with morphogens, growth factors, cytokines, cell receptors, cell adhesion molecules, and neurotrophic peptides to facilitate regulatory roles in embryonic neural development [19]. HA is the only non-sulfated GAG and is fixed in hyalectan CSPGs aggregates in perineural structures so that it has important biophysical properties which are important for the segregation and hydration of PNNs to provide neuroprotection and neural plasticity, as well as to regulate memory and cognition [19] (Figure 1).

**Figure 2:**
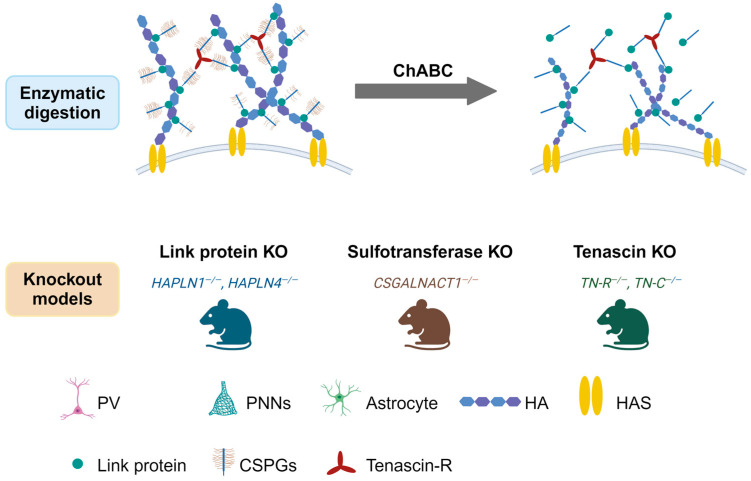
Summary of the current methods for modulating PNNs. PNNs can be modulated using several methods, including enzymatic degradation and genetic manipulation. Enzymatic degradation involves the use of enzymes, such as ChABC, to digest the glycosaminoglycan chains of PNNs, leading to their partial or complete removal (**Top**). Genetic manipulation techniques can be used to selectively delete or modify genes involved in PNN formation or maintenance, allowing for the study of specific molecular pathways (**Bottom**).

PNNs are formed by a cartilage-like structure as mesh-like webs, with link proteins (Crtl1/Hapln1 and Bral2/Hapln4) to stabilize the binding of various CSPGs to a HA backbone [20,21]. The CSPG components including lecticans, neurocan, versican, aggrecan, phosphacan, hyaluronan, tenascin-R, and link proteins, which are vulnerable to enzymatic degradation, primarily by chondroitinase-ABC (ChABC) [22]. Several animal KO and transgenic mouse models have been developed to prevent or reduce the formation of PNNs around neurons [23,24,25] (Figure 2). The cellular origin of CSPGs within PNNs has been a longstanding topic of debate. Investigations have shown that while certain CSPGs are produced and expressed by neurons, others have glial origins, and some even derive from both, ultimately converging into the extracellular space to assemble into PNNs [26,27,28]. In addition, aggrecan and neurocan are expressed by neurons, while brevican is expressed by both neurons and astrocytes [22]. Histologically, PNNs’ visualization is predominantly achieved through lectin wisteria floribunda agglutinin (WFA) staining, due to its affinity for N-acetylgalactosamine in the polysaccharide chain of most PNNs [29,30], although alternative methods have also been used [31,32]. It is worth noting that there are also WFA-negative PNNs. The PNNs that surround cortical output neurons do not bind to WFA and are instead recognized by aggregative proteoglycan antibodies [33].

PNNs play a key role in synaptic stabilization by acting as a physical barrier, as well as in the integration and generation of neuronal electrical activity. They provide a continuous microenvironment that facilitates the flow of cations across the membrane, thereby contributing to the electrical properties of neurons [34,35]. As mentioned earlier, the vast majority of the mammalian CNS cells are endowed with PNNs. Meanwhile, PNNs surround various types of nerve cell and exhibited a conserved distribution across mammalian species, from rodents to primates and human [36]. PNNs mostly surround the soma and dendrites of GABAergic neurons, especially around fast-spiking parvalbumin interneurons (PV cells) in various brain regions, like the forebrain, midbrain, and cerebellum [20,35,37,38,39]. Notably, PNNs envelop a smaller number of excitatory neurons [40], which can either express PV or not [41,42]. Furthermore, they also wrap around neurons pivotal to fast transmission, including glycinergic output neurons in the medial nucleus of the trapezoid body (MNTB) at the calyx of Held synapse, and excitatory neurons in the deep cerebellar nucleus [43]. In the hippocampus, PNNs are typically formed around inhibitory interneurons [44], yet in the CA2 region, PNNs have been observed surrounding both inhibitory and excitatory neurons [45]. Intriguingly, the expression of PNNs exhibit sexual dimorphism in certain regions. For instance, it has shown that PNNs are numerous and well developed in hippocampal CA1 of adult male rats but are lower in juvenile and possibly in adult females. Such differences, however, are not evident in other regions, like CA3 or the adjacent neocortex [46].

### 2.2. Physiological Functions

PNNs emerge during postnatal development, reaching its expression peaks in adulthood and presenting minimally in both newborn and aged animals [5]. The appearance of PNNs in juveniles coincides with the closing of critical developmental periods [2,47]. In the meanwhile, it is known that disruptions of either sulfate supply or sulfase can affect brain development and have long-lasting effects on brain function [48]. The principal reason affecting the CNS functions mentioned above is that PNNs strategically influence the development and stabilization of synaptic connections [49,50]. The functions of PNNs actually include various forms of plasticity. Numerous studies have shown that PNNs appear on PV inhibitory neurons, reaching mature levels by the end of the critical period during postnatal development. In adult animals, disrupted PNNs and associated signaling restore the visual cortical plasticity [2]. Fast-spiking, calcium-dependent, parvalbumin-positive (PV+) GABAergic interneurons are the most significant cells enwrapped by PNNs and they play an important role in maintaining plasticity and timing during critical periods [51]. When PNNs in sensory cortex are mature ahead of time, plasticity in the local network is greatly reduced. However, experimental removal of PNNs in adult animals can restore plasticity levels in young animals by increasing the structural plasticity and reducing the inhibitory spiking [2,52]. The correct pattern and assembly of PNNs is a key step in the maturation of PV+ GABAergic interneurons, and in the regulation of synaptic plasticity [53]. These finding suggest that the deposition of PNNs onto PV inhibitory neurons applied the brakes to critical period plasticity [54]. In this context, GABA antagonists reactivate the critical period plasticity and reduce the number of PNNs, whereas GABA agonists restore PV/PNN expression and limit plasticity in the aged cortex [55,56]. Mirroring these findings, chemogenetic inactivation of PV+ interneurons is sufficient to reinstate the critical period plasticity in the adult auditory cortex, and, in this process, PNNs undergo the same anatomical changes with PV+ [57]. Along with diminished neuronal and glial elements, Elise et al. (2021) found atypical PNNs that regulate plasticity in the CA2 of BTBR mice (social dysfunction) [58]. By diminishing PNNs in the CA2 of BTBR mice to control levels, they observed a partial restoration of social memory [58]. In conclusion, degradation of PNNs not only restores critical period plasticity but also modifies neuronal plasticity in response to a strong stimulus.

Structurally, PNNs restrict neurite growth and synapse development. At the synaptic level, PNNs compartmentalize the neuronal surface and restrict glutamate receptor mobility [59], supporting synaptic plasticity and stabilizing synapses. Preventing AMPA receptor mobility reduces short-term plasticity in rat primary neurons, indicating a potential role of PNNs in memory formation. Several PNN components regulate synaptic plasticity. Neurocan deficiency reduces late-phase LTP stability [60], brevican ablation significantly impairs LTP [61], and depletion of its binding partner tenascin-R also reduces LTP [62]. Knockout of the related glycoprotein tenascin-C results in a complete failure in LTD induction and impaired LTP development, likely due to reduced L-type VDCC channel signaling [63,64].

PNNs are crucial for protecting neurons from oxidative stress [23,65,66]. CS and HA are highly charged and rich in anions. Therefore, PNNs can bind a large number of cations than other ECM components, such as Fe^3+^, participate in local ion homeostasis maintenance, and reduce oxidative stress in the CNS [67]. Modified PNNs, stemming from exposure to a variety of substances, like high-fat diets [68,69] and cocaine, might impair cognition and memory [70,71]. For instance, a high-fat, high-sugar diet reduced PV neurons, and altered the coexpression of PNNs in adolescent rats, resulting in impaired social memory in the medial prefrontal cortex (mPFC) [69]. PNNs also have a neuroprotective function by protecting PV neurons from oxidative stress, because the PV neurons without a PNN structure are highly vulnerable to oxidative stress, as revealed by a previous study [72]. In particular, PV neurons are relevant to the physiological state of an increased metabolic activity rate and promote the production of reactive oxygen species. When the antioxidant function of PNNs are deficient, the excessive production of reactive oxygen in PV neurons leads to cell death [73].

PNNs have been implicated in controlling various forms of memory [30,74,75]. Inhibitory neurons, especially PV cells, across many regions play a pivotal role in shaping the memory engram [76,77,78,79]. Supporting this, disruptions in PNNs or their components has been observed in a number of psychiatric disorders related to learning, memory, and information processing [80]. It is now well established that PNNs ensure the stability of existing synapses and prevent the formation of new ones on mature PV interneurons [81]. As PNNs stabilize excitatory inputs, they lead to an increase in the maximum firing rate of PV interneurons, and in PV protein expression [44,82]. More recently, PNNs are shown to act to stabilize synaptic contacts, to limit further plasticity, to restrict the synaptic changes, and to help to solidify active synaptic networks, which are the basis for memory acquisition [81,83,84]. It has also been demonstrated that the absence of PNNs increases terminal axonal sprouting, synaptic plasticity, and memory retention in adult mice and restores memory in an Alzheimer’s model [71,85]. Post-learning sharp wave ripple (SWR) activity involves the sequential replay of training-related neuronal assemblies and is critical for system-level memory consolidation [86]. Sun et al. (2018) found that pretreatment with chondroitinase cleaved PNNs sidechains and increased SWR frequency [87]. Therefore, this reduction in PNNs would allow neurons to respond to changes in the environment. Indeed, degrading PNNs with ChABC promotes the assembly of new inhibitory synapses onto PV cells, which would promote new learning [88] and could even erase consolidated memories [89].

PNNs also regulate neural regeneration in adulthood by surrounding and stabilizing synaptic contacts. After brain injuries in mammals, a scar often forms, consisting of fibrous tissue in the lesion core and glial tissue, which creates a chemical barrier containing various ECM molecules [90,91]. Notably, these chemical barrier molecules, mainly CSPGs and CS-GAGs, limit the ability of axons to regrow over long distances and inhibit new contact with descending axons [91,92]. Previous studies revealed that the administration of ChABC after spinal cord injury removed CS-GAGs from PNNs at the lesion site, thus promoting plasticity and nerve regeneration in long tracts entering the lesion site. In goldfish with a spinal hemisection, plasticity in the spinal neurons might be restored by downregulation of CS in the PNNs in the regeneration process [93]. Moreover, the administration of ChABC degrades CSPGs in the PNNs and appears to boost the plasticity of spinal neurons and to promote the sprouting of spared long tract axons [94]. Nevertheless, it is important to note that thorough digestion of CSPG GAG chains through intracortical ChABC injection might worsen motor deficits and hinder axonal sprouting in the corticospinal tract above the lesion in spinal cord injury models. These findings indicate that CSPGs modulation requires meticulous control to yield functional benefits [95]. It is well-established that in the mature nervous system, motoneurons located in the ventral horn of the spinal cord are surrounded by structures known as PNNs [96]. Recent research suggests that preserving motoneuron PNNs and minimizing synaptic stripping through exercise could facilitate the maintenance of the spinal circuitry and have beneficial effects on functional recovery following peripheral nerve injury [97]. It suggests that PNNs in the spinal cord can be differentially regulated by peripheral injury and activity when compared to brain injury. Based on the aforementioned information, it is evident that regeneration in the CNS, or the absence thereof, is tightly regulated by PNN formation. In a word, PNNs are related to stabilizing synaptic connectivity and protecting neurons by forming a physical barrier. PNNs protect the integrity of synaptic junctions and insulate them from potentially damaging stimuli, such as oxidative stress. Regulation of PNNs could promote or inhibit synapse formation to modify the timing and precision of information processing, memory, and cognition [12].

## 3. Roles of PNNs in Learning and Memory

### 3.1. Object Recognition (OR) Memory

Object recognition (OR) memory refers to an organism’s ability to recognize and remember previously encountered objects as familiar, distinguishing them from novel objects. OR memory tests, often using rodents, assess cognitive function by measuring the time spent exploring novel objects versus familiar ones, with a preference for the novel indicating intact memory [98,99]. The removal of major structural components of PNNs in the perirhinal cortex—a brain region important for object recognition—via ChABC in two mouse models exhibiting a significant impairment in OR memory effectively restores their OR memory and synaptic transmission to levels comparable to normal mice after one week [71,85]. A study emphasized the functional significance of neurocan in supporting perisomatic GABAergic inhibition, temporal order recognition memory, and cognitive flexibility. These functions are crucial cognitive resources that are often depleted in neuropsychiatric disorders [100]. Similarly, depletion of PNNs in the perirhinal cortex increases long-term object recognition memory as measured by spontaneous object recognition [85]. Systemic oral administration of 4-methylumbelliferone (4-MU) for 6 months reduces PNN formation around neurons and enhances memory retention in mice in spontaneous object recognition test [101]. Furthermore, downregulation of PNNs either through ChABC digestion or genetic knockdown can improve object recognition memory for a period up to 48 h [102]. PTPσ+/− mice, having a lower number of PNN receptors, display a disrupted PNN network consolidation function in their brain, which bolsters their short-term memory performance but impairs the long-term memory performance [103]. Interestingly, the cognitive and behavioral effects of ChABC vary depending on the location and time of treatment [104]. For example, the infusion of ChABC into the perirhinal cortex enhances recognition memory in an object recognition paradigm. However, the memory-enhancing effect of ChABC treatment attenuates overtime, suggesting that the regeneration of PNNs gradually restores the levels of plasticity [85]. In contrast, infusion of ChABC into the mPFC impairs cross-modal object recognition and object oddity tests in rats [105]. Carulli et al. (2020) [106] provided evidence of dynamic modulation of PNNs in response to delayed eyeblink conditioning (EBC), which is an associative learning paradigm.

Modulation of PNNs could change the balance between excitatory and inhibitory inputs to deep cerebellar nuclei (DCN) neurons which regulate the acquisition and retention of memory [106]. In addition, it has been known that PV interneurons, which predominantly contribute to the generation of GBO, are surrounded by PNNs [107]. Vitamin D deficiency (VDD) could increase the spontaneous GBO and decrease the evoked GBO, reminiscent of the aberrant GBO in schizophrenia, and, as such, decrease PNNs via this process [108]. Moreover, patients with depression often also suffer from cognitive impairments. Short-term object recognition memory was decreased, and PNNs’ expression was increased in the hippocampal CA1 region of depressed rats. Intracranial injection of ChABC into hippocampus CA1 could restore PNNs expression, LTP, hippocampal inhibitory tone, and memory performance in object recognition tests [109]. Therefore, enzymatic digestion of PNNs improves learning, but intact PNNs are necessary for memory retention.

### 3.2. Fear Memory

Fear conditioning induces a permanent memory in adult animals, often examined by training an animal to associate the presentation of a cue, typically a tone, with a footshock [110]. In early postnatal development, especially in rats younger than 3 weeks, extinction of conditioned fear leads to memory erasure. In contrast, in adult animals, extinction training creates a new memory but does not erase the original memory, suggesting that fear memories are actively protected in adults [89]. This protection is conferred by CSPGs, components of PNNs. The presence of PNNs enables original fear memory and extinction memories to coexist, protecting the former from erasure. Injection of ChABC in adult mice before acquisition of fear memory makes the rate of fear extinction similar to that observed in juvenile mice, indicating a greater response to extinction training. However, injection prior to extinction but after fear training was ineffective. Therefore, PNNs may be involved in the initial encoding process of fear memory and reinforcing their resistance to extinction, rather than in directly regulating the extinction process [111]. ChABC-dependent degradation of PNNs promoted the elimination of fear memory, underscoring the role of intact PNNs in erasure-resistant fear memories [89]. Accordingly, the transplantation of immature interneurons reduced the expression of PNNs and offered an expanded capacity of plasticity in response to the facilitation of extinction memory [112].

Beyond memory extinction, memory encompasses the processes of formation, consolidation, and reconsolidation [37,113]. PNNs have also been implicated in these three processes, fundamentally rooted in neural plasticity. The acquisition of auditory fear memory is associated with an increase in PNNs in such regions as the hippocampus, auditory cortices, and anterior cingulate cortex. Reinhard et al. (2019) found impaired tone-associated fear memory formation in the mouse model of FXS (Fragile X syndrome, Fmr1 KO mice), and this was paralleled by impaired regulation of PNNs in the superficial layers of auditory cortex in Fmr1 KO mice [114]. The elimination of PNNs impairs the inhibitory PV neurons, which are critical for the consolidation of auditory fear memories [115]. However, in the amygdala, PNNs were mainly expressed around excitatory neurons, which were recruited during auditory fear conditioning and memory retrieval [70]. Intriguingly, Evans et al. (2022) conducted post-learning manipulation of neurogenesis through voluntary exercise, and observed a reduction in PNN density in the CA1, accompanying a decrease in contextual fear memory retrieval [116]. More studies focus on the role of PNNs in memory consolidation and retention. Increased PNN expression in both the hippocampus and the ACC enhances the recall and reconsolidation of both recent and remote fear memory, whereas removal of PNNs impairs the consolidation and reconsolidation of both recent and remote fear memory by regulating the feedback inhibition of PV interneurons [37]. However, digestion of PNNs using ChABC in the secondary visual cortex (V2L) interrupts the recall of long-term fear memory but not recent fear memory in rats [117]. Several studies prior to this work also showed similar remote fear memory recall impairment when PNNs were disrupted in various regions of the brain [89,118]. Similarly, injection of hyaluronidase in the hippocampus to destroy another ingredient of PNNs attenuated fear responses one day after conditioning, and a combination of hyaluronidase plus ChABC injected into the hippocampus also attenuated long-term fear memory [118,119]. The elimination of PNNs in CA1 disrupted GABA release and long-term contextual fear memory retention [120]. Jovasevic et al. (2021) demonstrated that depleting primary cilia in the hippocampal CA1 subfield was accompanied by the disruption of PNNs in CA1 and played an important role in the persistence of fear memory. Therefore, the severe downregulation of the primary cilium-associated genes, as well as several genes encoding important component of PNNs, may lead to the failure of lasting memory [121]. Intriguingly, diurnal and circadian rhythms of PNNs were found in several brain regions involved in emotional memory processing. For example, sleep deprivation prevented the daytime decrease in PNNs in the hippocampus, BLA, and central amygdala (CeA) and enhanced fear memory extinction. Thus, rhythmic modification of PNNs may contribute to memory consolidation during sleep [122]. Collectively, PNNs play an important role in many facets of fear memory, and this functionality relies on myriad mechanisms that appear to be rhythmic and brain region-dependent.

### 3.3. Spatial Memory

The maturation of PNNs in the local network is known to greatly reduce the plasticity of the sensory cortex. It is interesting to note that the development time of PNNs coincides with the development of grid cell firing, which is an essential part of the network supporting navigation and spatial memory [123]. Once the critical period is over, PNNs play a crucial role in maintaining the stability of established synaptic connections, thereby ensuring both the network’s integrity and the spatiotemporal interplay among grid cells [124]. This stability is particularly important in maintaining consistent grid cells when revisiting previous environments [124]. Neuropeptide Y (NPY) is involved in regulating various physiological functions, including learning and memory abilities. Studies have demonstrated that Npy1rrfb mutant mice exhibited a significant slowdown in spatial learning, which was associated with an intense increase in PNN expression. Interestingly, enzymatic digestion of PNNs restored their learning abilities [125]. Tajerian et al. (2018) reported aberrant hippocampal LTP and a reduction in specialized PNNs around inhibitory interneurons, correlating with deficits in location memory [126]. Iron is a necessary substrate for neuronal function throughout life. Iron deficiency can impair spatial memory and result in disorganized apical dendrite structure accompanied by altered PV and PNN expression, along with reduced BDNF levels [127]. These findings suggest the crucial roles of PNNs in maintaining plasticity in the sensory cortex and spatial memory and highlight the potential for interventions targeting PNN expression to improve cognitive function.

## 4. Roles of PNNs in Memory-Related Dysfunctions

### 4.1. Addiction

Drug addiction modifies brain plasticity, leading to persistent drug reward memory. These alterations in plasticity and memories are believed to produce aberrant motivation and reinforcement, contributing to addiction. While much research has focused on the effect of drug abuse on pre- and postsynaptic cells and astrocytes [110], recent studies have highlighted PNNs and their individual constituents as important regulators of memories linked to addiction-related behaviors in animals. Emerging evidence, including our own, has shown that degradation of PNNs affects extinction and attenuates cocaine-conditioned place preference (cocaine-CPP) and cocaine self-administration [128,129]. PNNs were altered in specific brain regions when exposed to several drugs of abuse (cocaine, heroin, nicotine, and alcohol) [129]. Cocaine induces neuroplasticity in the ECM, evident in both cocaine-dependent humans [130] and rodent models of cocaine addiction [131]. Meanwhile, cocaine also induces metaplasticity [132], which alters the ability of natural stimuli to further alter plasticity. In this case, the removal of PNNs and/or regulation of other PNN-binding molecules may alter cell firing and homeostatic plasticity [133] so as to prevent the drug-induced changes, possibly by restoring the excitatory/inhibitory balance [65].

Modulation of the expression of PNN components seems to be influenced by the type of drug, the duration of drug exposure and withdrawal, and the brain region [129]. Key areas, like the BLA, hippocampus, and prefrontal cortex (PFC), played a critical role in acquiring and maintaining the drug-related memory. These regions notably overlap with brain regions essential for fear-conditioned memory [134]. Golgi neurons are vital for the modulation of activity and plasticity in the cerebellum, and PNNs surrounding Golgi interneurons play a role in consolidating drug-related memories [135]. Fully condensed PNNs around Golgi interneurons can label synaptic alignments that represent drug-cue associations [135]. A recent study showed that cocaine-induced conditioned preference increased neural activity and upregulated PNNs around Golgi interneurons in the posterior cerebellar cortex. However, a reduction in PNN protein expression around Golgi cells disrupted the consolidation process [20]. The inactivation of the IL (infralimbic cortex), but not the PrL (prelimbic cortex), increased posterior cerebellar cortex activity, and upregulated PNNs’ expression around Golgi interneurons. This can further intensify the preference for cocaine-related cues and the acquisition of cocaine-induced memory [136]. PNNs within the mPFC are also required for the maintenance of cocaine-associated memories [137], such as when PNN removal in the mPFC attenuated the acquisition and reconsolidation of cocaine-associated memory in a CPP task [138]. Cocaine memory reactivation significantly altered the synaptic and electrical properties of the PNN+ neuron in mPFC and its related proteins that regulate pyramidal output and influence the drug-seeking behavior [139]. Removed PNNs within the BLA or CeA enhanced extinction training and changed plasticity prior to extinction training of morphine or cocaine-induced CPP and heroin self-administration [140]. Increased densities of hippocampal PNNs, coupled with decreased ECM proteolytic genes and altered synaptic markers, support preclinical studies indicating that PNNs may stabilize reward memories. PNNs and ECM molecules may be promising targets for addressing cue-induced relapse in SUD [141]. In summary, PNN removal appears to create a blank slate for neuroplasticity that has the potential to combat drug-induced maladaptive plasticity and create new adaptive response, suggesting that targeting PNNs may be promising for the treatment of addiction.

### 4.2. Aging

As the nervous system ages, there is a gradual decline in its plasticity, as evidenced by the reduced capability of the CNS in the ability of learning, as well as the decrease in the ability to adapt to environmental changes and compensate for damage. Elevated microglial activity in aged brains negatively impacts cognition, in part through mechanisms that alter PNN assembly in memory-associated brain regions [142]. As mentioned before, CSPGs in PNNs are central to regulating synaptic plasticity [143]. Thus, PNNs have crucial roles in memory in the aging brain, and deterioration of PNNs contributes to age-dependent brain dysfunction [19]. The accumulation of CSPG-associated ECM correlates with age-dependent decline in striatum-related cognitive functions, including motor learning and working memory. Removal of CSPG-associated ECM by ChABC in aged mice significantly improved motor learning, suggesting that changes in the neural ECM’s CSPGs can regulate striatal plasticity [144]. The CSPGs of PNNs also have important functional roles in protecting against the development of AD. Cortical regions with abundant levels of ECM CSPGs seem to be less vulnerable to degenerative features associated with the development of AD [145,146]. The decline in PNNs, marked by the loss of lectin labeling of the PNN-associated CS-GAGs [147,148], has been reported in patients with neurocognitive and neuropsychiatric disorders, including AD [149]. Furthermore, PNN-associated CS-GAG sulfation patterns are also altered in AD brains [150,151]. Brain-specific lectican brevican were significantly elevated in AD patients, which suggests the loss of synaptic plasticity before cell death.

PNNs seem to provide protection against the formation of neurofibrillary tangles [152]. The colocalization of some proteoglycans with Aβ and tangles may reflect a pathogenic role of the PNNs in AD [153]. Consistently, it has been shown that disordered quantification of PV and PNNs in the hippocampus of Tg2576 mice are early events of amyloid pathology [154]. The application of ChABC significantly reduce amyloid β-peptide (Aβ) burden and increase synaptic density [155]. When ChABC is injected into the perirhinal cortex to digest the existing PNNs, the Tau mice show OR memory and synaptic transmission comparable to those of control animals, suggesting that PNNs is related with memory loss in neurodegenerative disorders, like AD [71]. Of the two mono-sulphated CSs [156], C6S is known to facilitate axon growth and plasticity, while C4S’ influence is the opposite [157]. In AD animal models, using a C4S-blocking antibody have been shown to alleviate the pathology-associated memory loss [71,158]. A decline in C6S is linked to premature memory loss, while an increase in C6S not only prevents age-related memory deficits but can also restore existing memory impairments [159]. An enriched environment might improve cognition in AD mouse models by modulating the PNN levels, which has been proven to have long-lasting beneficial effects on memory in AD subjects [154]. In conclusion, the change in PNN GAG sulfation renders the PNNs more inhibitory, which leads to a decrease in plasticity and adversely affects memory in aged brains [156]. By modulating PNNs, it is possible to restore neural plasticity to a juvenile-like state, which results in the restoration of cognition in both AD-affected and aged mouse models [14,102,158].

### 4.3. Other

Consistent with working memory impairments in schizophrenia, multiple post-mortem studies have demonstrated morphological and/or molecular alterations in the dorsolateral prefrontal cortex (DLPFC) layer 3 pyramidal cells (PCs) and PV neurons in individuals with schizophrenia [160]. These alterations include reduced dendritic spine density on PCs, lower levels of PV mRNA, and reduced PV protein in PV basket cell terminals. The lower levels of PV mRNA and protein could be explained by a loss of PV neurons [161]. However, studies investigating PV cell density in schizophrenia have provided mixed results. A lower density of perineuronal nets (PNNs) in schizophrenia could result from several different pathological processes. Firstly, it could be the result of a lower density of PV neurons for PNNs to surround. Secondly, fewer PNNs could indicate a failure in PNN formation and/or maintenance, which would be characterized by a loss of multiple PNN markers and a lower proportion of PV neurons surrounded by PNNs, without a reduction in PV cell density. Thirdly, the detectability of PNNs could be reduced due to alterations in their composition, such as lower glycosylation of aggrecan. Given the roles of PNNs in regulating PV cellular physiology, the identified alterations in PV neurons and their PNNs could contribute to dysfunction in schizophrenia [162]. In Fmr1 knockout (KO) mice, a model for autism, there is a reduced density of PNNs in the amygdala and auditory cortex under all conditions. Additionally, there is a decrease in PNN intensity in the CA2 region of the hippocampus. Importantly, there appears to be a positive correlation between tone-associated memory and the density of PNNs in the amygdala and auditory cortex [114].

## 5. The Challenges and Prospects

Recent work has revealed the importance of PNNs in the control of CNS plasticity [19]. The removal of PNNs provides an opportunity to alter plasticity for CNS repair after injury and to facilitate learning and memory in aging and CNS disorders. However, the therapeutic potential of targeting PNNs remain in its nascent stage.

Firstly, the current understanding on the roles of PNNs in memory and associated psychiatric conditions has been developed through a large number of behavioral studies involving proteolytic cleavage and knockout models [163]. Genetic modifications, like KO models, are frequently used to abolish or attenuate PNNs. While these methods are very useful to establish the proof of concept that modulating PNNs can profoundly influence plasticity and memory, their direct clinic application remains challenging. Future studies should examine the potential genetic associations of other genes encoding PNN components in memory [129]. For more refined alteration in neuronal plasticity, strategies that specifically target and modify the molecular composition of PNNs are essential [164].

Secondly, the connection between PNNs and the modulation of learning and memory primarily revolve around four genes encoding PNNs components: Hapln1, TnR, TnC, and Bcan. However, the involvement of these and other PNNs components in long-term memory mechanisms is largely unclear and is currently a focus of research [165]. A majority of studies used a global disruption method, targeting not only the PNNs, but also the entire ECM and other neurons. Future studies need more specific approaches to target the PNNs and to verify the effects of these studies [110].

Thirdly, there is ambiguity surrounding the specific cell types responsible for in the production of PNNs [165]. For instance, astrocytes are able to package HA-based pericellular matrices in vitro [166], whereas CSPGs, such as aggrecan, brevican, and phosphacan, are produced by neurons [167]. Intriguingly, other evidence suggests that the neurons themselves seem to be able to create a PNN-like structure in a dissociated culture in the absence of glial cells [167].

Lastly, it is imperative to resolve how the PNNs actually affect the activity of individual neurons in the neuronal system. This necessitates in vivo electrophysiological signals or image calcium perfusion to distinguish between the cells that are enwrapped by PNNs and those that are not. However, a significant challenge is the current lack of tools for the in vivo staining of PNNs [168]. It is also worth noting that manipulation of PNNs probably causes neuronal circuits to experience hyperplasticity or might affect synapses vulnerable to neurotoxic stimuli, which could have a noxious effect on cognition [12]. While PNNs may not supersede neurons as the primary entity of research, their growing prominence in decoding brain functions and therapeutic potential cannot be overlooked [104].

## 6. Conclusions

In this review, we have explored the intricate molecular compositions and functions of PNNs. The emerging evidence suggests that PNNs have a vital role in controlling diverse facets of memory. Notably, PNNs appear to influence the capacity for new learning and/or memory formation, especially during both extinction and reversal learning, by restricting the critical-period plasticity. PNNs are also implicated in a spectrum of neuropsychiatric disorders related to memory, encompassing schizophrenia, drug addiction, and neurodegeneration. As we look forward, it is crucial for future studies to discover the detailed dynamic molecule changes occurring in PNNs in order to understand how PNNs and PNN-surrounded neurons influence memory processes. In conclusion, the diverse protein elements of PNNs present promising therapeutic avenues, potentially for aiding in erasing detrimental memories that drive relapses into drug abuse, rejuvenating neural plasticity and restoring cognition in neurological disorders, like AD.
